# Causal relationships between psoriasis and coronary artery disease: A two-sample Mendelian randomization study

**DOI:** 10.1097/MD.0000000000047503

**Published:** 2026-02-06

**Authors:** Yang Xu, Juhua Zhao

**Affiliations:** a Beijing Anzhen Nanchong Hospital of Capital Medical University & Nanchong Central Hospital, Nanchong, Sichuan, China.

**Keywords:** coronary artery disease, Mendelian randomization, psoriasis

## Abstract

Previous studies have revealed a potential association between psoriasis and coronary artery disease (CAD). However, the causal relationship between the 2 remains unclear. This study aims to assess the causal link between psoriasis and CAD, which encompasses coronary heart disease, myocardial infarction, and angina pectoris. After obtaining genome-wide association studies data on psoriasis and CAD, we selected appropriate single nucleotide polymorphisms for Mendelian randomization (MR) analysis. The inverse-variance weighted method was used as the main analytical method. The inverse-variance weighted method indicated that psoriasis was associated with a higher risk of CAD (odds ratio = 11.538, 95% confidence interval = 4.498–29.595, *P* < .001), and coronary heart disease (odds ratio = 1.080, 95% confidence interval = 1.031–1.132, *P* = .001). Reverse MR analyses did not show causal effects of CAD on psoriasis. This study shows a significant causal association between psoriasis and incidence of CAD. However, there is no reverse causal association between CAD and psoriasis.

## 1. Introduction

Psoriasis is a chronic, immune-mediated inflammatory skin disease that significantly impairs the quality of life of affected individuals.^[1]^ The global prevalence of psoriasis is approximately 2% to 3%, significantly affecting the normal life and psychological state of patients.^[2,3]^ Patients with this condition frequently experience comorbidities associated with other systemic diseases, including cardiovascular diseases, mental health disorders, autoimmune diseases, and metabolic syndrome. Consequently, psoriasis is often classified as a systemic disease.^[4,5]^ Coronary artery disease (CAD) is a common cardiovascular condition that includes various forms, such as coronary heart disease (CHD), myocardial infarction (MI) and angina pectoris, and has become a major risk factor for mortality globally.^[6,7]^

Psoriasis is widely recognized as a condition closely linked to cardiovascular diseases, with numerous studies demonstrating that it elevates the risk of developing CAD.^[8–10]^ A meta-analysis indicated a prominent association between psoriasis and the incidence of MI, CAD, and aortic aneurysm.^[11]^ CHD is a common form of coronary artery disease, and a large number of studies have identified a significantly higher incidence of CHD among individuals with psoriasis.^[12–14]^ However, a retrospective study found that non-psoriasis patients exhibited more severe manifestations of CHD than psoriasis patients with comparable clinical characteristics.^[15]^ Psoriasis appears to exert a protective effect against CHD. Furthermore, prior research has shown that psoriasis is associated with an increased risk of MI.^[16,17]^ In contrast, the findings of a large-scale cohort study indicated that psoriasis does not significantly elevate the risk of MI.^[18]^ The association between psoriasis and CAD remains controversial.

Mendelian randomization (MR) analysis is a widely utilized method for investigating causal relationships between exposure factors and outcome variables.^[19]^ MR employs genetic variants as instrumental variables, effectively mitigating potential bias resulting from confounding factors.^[20,21]^ This study aims to elucidate the potential causality between psoriasis and CAD through a 2-sample MR analysis.

## 2. Methods

### 2.1. Study design

**Figure 1.**
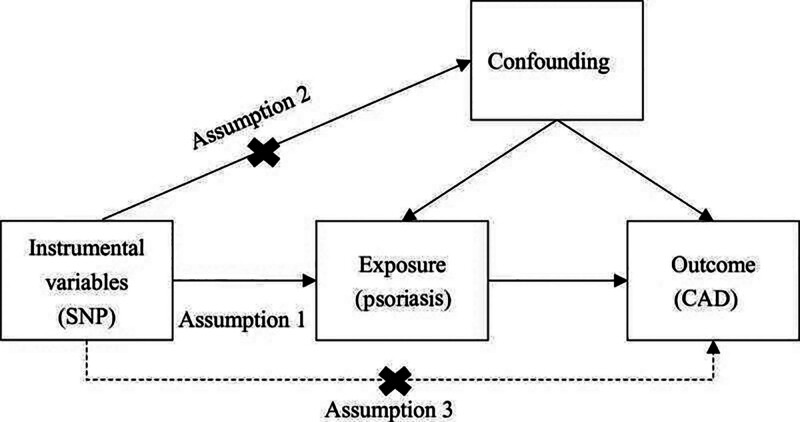
A flowchart of the causal association between psoriasis and CAD. CAD = coronary artery disease.

The data were derived from the summary statistics of genome-wide association studies (GWAS). This analysis necessitated the fulfillment of 3 primary assumptions: Exposure factors are strongly correlated with instrumental variables (IVs); There are no known confounding factors associated with either exposure or outcome; and IVs only affect outcomes when they are associated with exposure factors (Fig. 1).

### 2.2. Date sources

The GWAS data of psoriasis were sourced from the GWAS database, including 5427 cases and 479,171 controls. The GWAS data of CHD were derived from the GWAS database, including 10,157 cases and 351,037 controls. The GWAS data of CAD were sourced from the study by Mbatchou et al, which included 352,063 individuals.^[22]^ The GWAS data of MI and angina pectoris were derived from the FinnGen research consortium, including 12,801 cases and 187,840 controls for MI, and 18,168 cases and 187,840 controls for angina pectoris. The detailed characteristics of the dataset are presented in Table S1, Supplemental Digital Content, https://links.lww.com/MD/R330.

### 2.3. Selection of IVs

In this study, we selected a *P*-value of 5 × 10^−8^ as the threshold to screen out single nucleotide polymorphisms associated with psoriasis.^[23]^ Furthermore, the analysis of linkage disequilibrium was conducted using the following threshold (*r*^2^ < 0.001, kb = 10,000).^[24]^ The palindromic single nucleotide polymorphisms were excluded, and the IVs was assessed using the *F*-statistic (*F* > 10) to ensure that the exposure factors were strongly related to the obtained IV. We harmonized and integrated the selected datasets of exposure and outcome to guarantee that the effect alleles belonged to the same allele and eliminated palindromic SNPs .^[25]^ In addition, reverse MR analysis is conducted following the aforementioned steps.

### 2.4. Statistical analysis

In this study, we conducted a 2-sample MR analysis to assess the causal relationship between psoriasis and CAD. The inverse-variance weighted method was used as a major analytical method, complemented by 4 additional methods: MR-Egger regression, simple mode, weighted median, and weighted mode approaches.^[26]^ The Cochran *Q* test was used to identify heterogeneity (*P* > .05 indicated the lack of pleiotropy).^[27]^ Furthermore, horizontal pleiotropy was assessed using MR-Egger regression and MR pleiotropy residual sum and outlier (*P* > .05 indicated the lack of horizontal pleiotropy).^[28]^ The leave-one-out method is employed to assess the stability of MR results. We investigated the impact of individual SNPs on the MR results by sequentially removing each SNP.^[29]^ All MR analyses were conducted using the “TwoSampleMR” and “MRPRESSO” packages in R software (version 4.4.1). After applying the Bonferroni correction, results with *P*-values less than .0125 (0.05/4) were deemed statistically significant.^[30]^

### 2.5. Reverse MR analysis

To investigate the effects of different types of CHD on psoriasis, we conducted a reverse MR analysis to explore the reverse causal relationship between psoriasis and CHD.^[31]^ The specific reverse MR analysis methods was consistent with the forward Mendelian analysis.

## 3. Results

### 3.1. Causal effect of psoriasis on CAD

**Figure 2.**
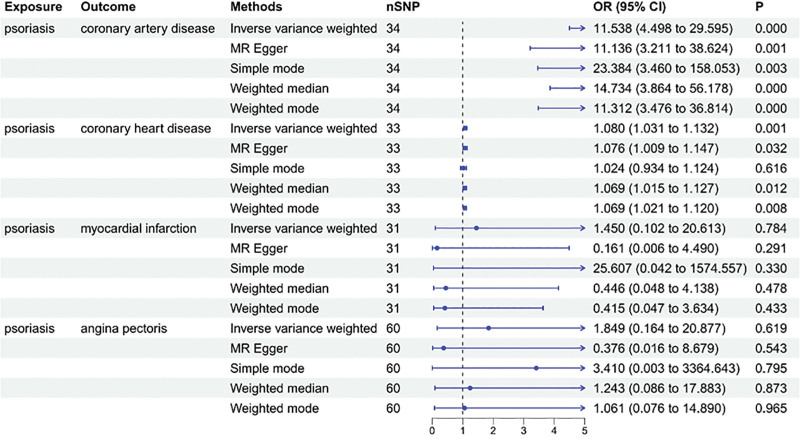
Association of psoriasis with the risk of CAD in MR analyses. CI = confidence intervals, CAD = coronary artery disease, MR = Mendelian randomization, OR = odds ratio.

The MR analysis revealed that psoriasis is associated with an increased risk of CAD and CHD (odds ratio [OR] = 11.538, 95% confidence interval [CI] = 4.498–29.595, *P* < .001; OR = 1.080, 95% CI = 1.031–1.132, *P* = .001). However, the MR analysis did not support significant associations with MI (OR = 1.450, 95% CI = 0.102–20.613, *P* = .784), angina pectoris (OR = 1.849, 95% CI = 0.164–20.877, *P* = .619) (Fig. 2) (Tables S2–S5, Supplemental Digital Content, https://links.lww.com/MD/R330).

### 3.2. Causal effect of CAD on psoriasis

**Figure 3.**
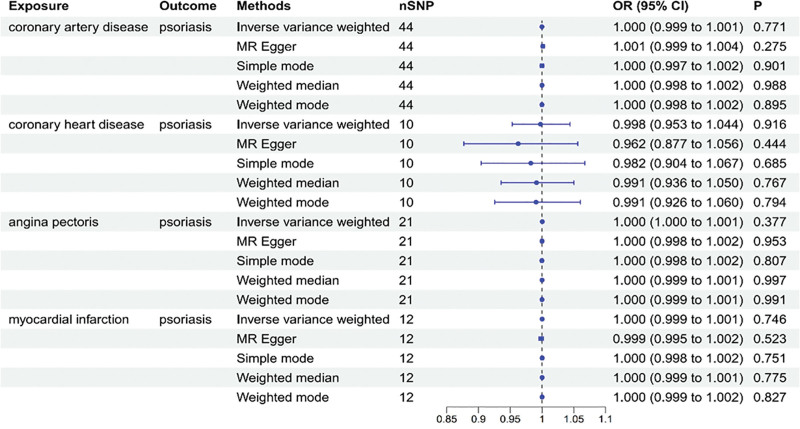
Association of CAD with the risk of psoriasis in MR analyses. CI = confidence intervals, CAD = coronary artery disease, MR = Mendelian randomization, OR = odds ratio.

The findings from the reverse MR analysis indicated no significant correlation between the risk of CAD and the occurrence of psoriasis (Fig. 3) (Tables S6–S9, Supplemental Digital Content, https://links.lww.com/MD/R330).

### 3.3. Sensitivity analysis

The MR-Egger regression and MR pleiotropy residual sum and outlier indicated no significant horizontal pleiotropy (*P* > .05). Inverse-variance weighted and MR-Egger results indicated the absence of heterogeneity (*P* > .05) (Tables S10 and S11, Supplemental Digital Content, https://links.lww.com/MD/R330). This suggests that our findings were generally not influenced by horizontal pleiotropy or heterogeneity. The leave-one-out analysis indicated that excluding any IVs did not alter the results, demonstrating the robustness of this MR analysis. The leave-one-out analysis, scatter plots and funnel plots also supported these findings (Figures S1–S8, Supplemental Digital content, https://links.lww.com/MD/R331).

## 4. Discussion

This study utilized a bidirectional 2-sample MR approach to explore the causality between psoriasis and CAD. The findings revealed that psoriasis may be a potential risk factor for CAD and CHD. Conversely, the results revealed that there is no significant causal association between psoriasis and the incidence of MI or angina pectoris. In addition, the reverse analysis revealed no significant association between CAD and psoriasis.

A large-scale retrospective study demonstrated that patients with psoriasis have a significantly elevated risk of developing CHD.^[13]^ Psoriasis is an independent risk factor for cardiovascular diseases. Patients with psoriasis exhibited a significantly elevated risk of developing CAD, with the risk further increasing in cases of more severe CAD.^[32]^ Another study suggested that psoriasis significantly increases the risk of developing CAD.^[33]^ Hjuler et al showed that psoriasis not only increases the risk of CAD, but also elevates the likelihood of developing severe CAD.^[34]^ Furthermore, Białecka et al found that patients with psoriasis have a significantly increased risk of concurrent atherosclerosis.^[35]^ Coronary artery calcification (CAC) is an independent risk factor for atherosclerotic cardiovascular disease.^[36]^ Moreover, the risk of atherosclerotic cardiovascular disease increases with higher CAC scores. Tinggaard et al proposed that psoriasis is associated with an increased risk of CAC.^[37]^ A clinical study indicated that patients with psoriasis exhibit a significantly higher prevalence and severity of CAC.^[38]^ Meanwhile, the results of a meta-analysis indicated a strong association between psoriasis and CAC.^[39]^ Numerous prior studies have demonstrated that psoriasis is a significant risk factor for CAD. Therefore, a comprehensive assessment of the physical condition of patients with a longstanding history of psoriasis is essential. Strict control of cardiovascular risk factors, including hypertension, hyperlipidemia, and hyperglycemia, is also necessary. When appropriate, novel therapeutic agents such as biologics should be employed to suppress the inflammatory response associated with psoriasis, thereby reducing the risk of adverse CHD events.

The pathogenesis of psoriasis and CAD is multifaceted, and the mechanisms underlying their association remain elusive. Atherosclerosis is the main pathogenic mechanism of CAD.^[40]^ Psoriasis and atherosclerosis exhibit similar pathophysiological mechanisms and express common inflammatory mediators and immune cells, including interleukin-2, tumor necrosis factor-α (TNF-α), vascular endothelial growth factor, chemokines, helper T cells, and NK cells.^[41,42]^ Both psoriasis and atherosclerosis exhibit shared characteristics, including the infiltration of T cells, monocytes, dendritic cells, and mast cells.^[43]^ Wang et al demonstrated that the psoriasis mouse model exhibited significant aortic inflammatory infiltration, accompanied by aortic thrombosis. Skin inflammation mediated by cytokines, such as interleukin-17 and TNF-α, promotes vascular inflammation and thrombosis.^[44]^ The combined action of exogenous interferon-gamma and TNF-α amplifies the inflammatory response in vascular endothelium and atherosclerotic lesions.^[45]^ Pro-inflammatory cytokines, such as interferon-gamma and TNF-α, serve as key mediators connecting psoriasis and atherosclerosis. An imaging study revealed increased inflammation in the skin, joints, and blood vessels of patients with psoriasis, with a notable elevation in aortic vascular inflammation.^[46]^

Psoriasis is closely associated with obesity and dyslipidemia. Adipose tissue secretes various inflammatory mediators that contribute to pathological alterations in the cardiovascular system.^[47]^ An increase in low-density lipoprotein content stimulates the oxidative reactions of activated macrophages, resulting in the generation of numerous foam cells. Monocytes adhere to activated endothelial cells, leading to an increased production of pro-inflammatory cytokines and chemokines, which subsequently contribute to the formation of atherosclerotic plaques. The pro-inflammatory cytokines produced by atherosclerotic plaques and adipose tissue exert effects on the liver, causing an elevation in C-reactive protein levels, a characteristic marker of cardiovascular diseases.^[48,49]^ A study examining the psoriasis genome identified functional pathways linked to skin lesions, metabolic disorders, and cardiovascular risks, which were overexpressed in serum.^[50]^ The findings suggest a potential association between altered gene transcription in the skin and common comorbidities in individuals with psoriasis. Atherosclerosis shares immunopathological features with psoriasis, notably the involvement of type 1 T helper cells.^[51,52]^ The T-cell subtypes implicated in psoriasis pathogenesis also contribute to atherosclerosis development.^[53,54]^ Dysfunction of immune cells represents a common pathogenic mechanism underlying both conditions.^[55]^

This study explored the causality between psoriasis and CAD through MR analysis. However, this study has several limitations. Firstly, since the GWAS data is derived from the European population, the applicability of the findings to other ethnic groups requires further verification. Additionally, while this study infers a causal relationship between psoriasis and CAD, further in-depth exploration of the interaction mechanisms between these 2 diseases is necessary. Consequently, the absence of detailed classification data limits our capacity to perform stratified analyses aimed at elucidating the causal relationships among various subtypes of the 2 diseases, including 4 subtypes of psoriasis: vulgaris, erythrodermic, pustular, and psoriatic arthritis.

## 5. Conclusion

Our research results indicate a positive causality between psoriasis and CAD. The findings of this study offer novel evidence supporting a genetic correlation between the 2 diseases. Therefore, early screening for CAD should be conducted in the psoriasis patient population, and preventive measures should be implemented to mitigate the risk of developing CAD.

## Acknowledgments

The authors thank the genome-wide association study for sharing public data.

## Author contributions

**Data curation:** Yang Xu.

**Formal analysis:** Yang Xu.

**Investigation:** Yang Xu.

**Writing – original draft:** Yang Xu.

**Writing – review & editing:** Juhua Zhao.

## Supplementary Material




