# Supplementary material for: Causal relationships between psoriasis and coronary artery disease: A two-sample Mendelian randomization study
Source: Medicine (Baltimore). 2026 Feb 6;105(6):e47503. doi: 10.1097/MD.0000000000047503 (PMC12885691; doi:10.1097/MD.0000000000047503)
Supplement: Supplementary file 2 [file medi-105-e47503-s002.docx]

**A**

**
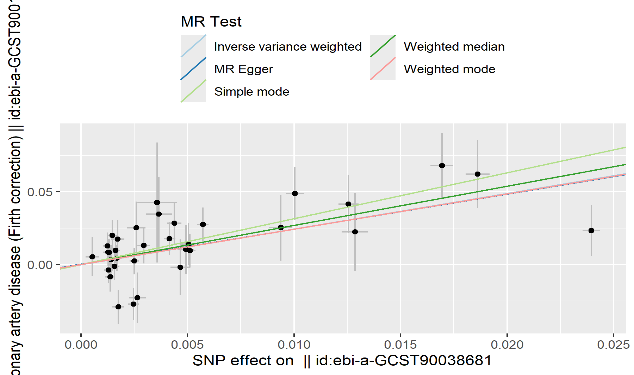
**

**B**

**
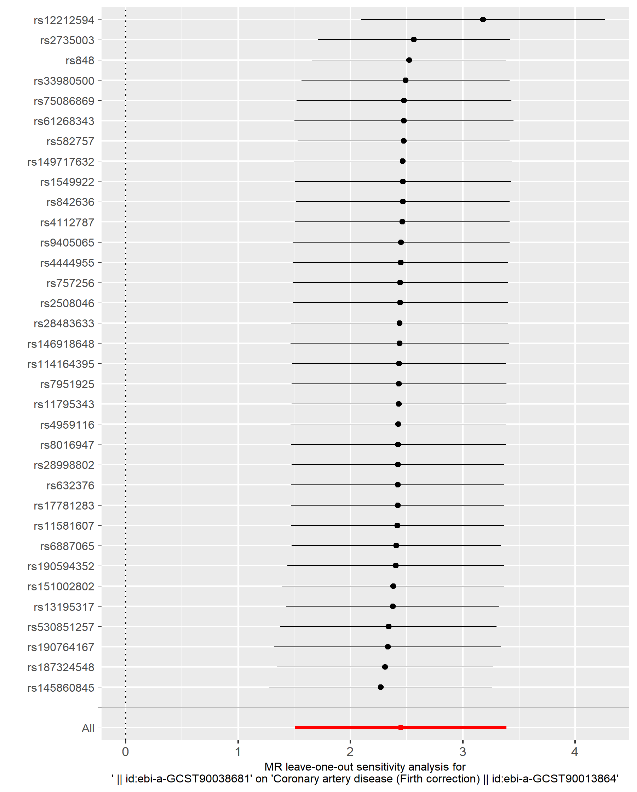
**

**C**

**
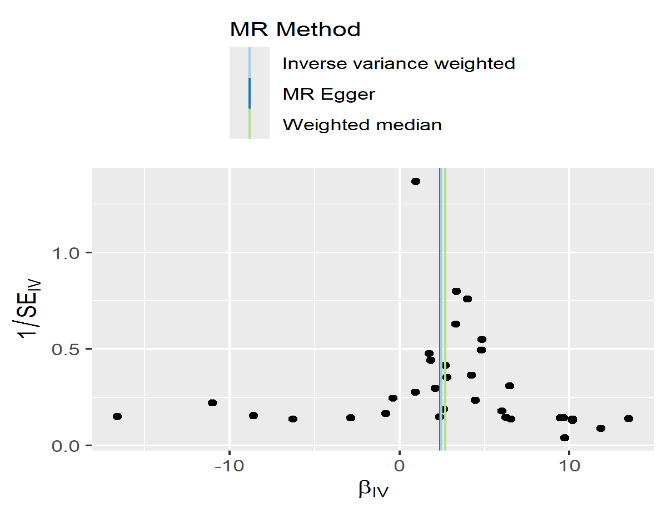
**

**Figure S1.** Scatter plots (A), Leave-one-out analysis (B) and Funnel plots (C) of psoriasis on CAD.

MR: Mendelian randomization; SNP: Single Nucleotide Polymorphism; CAD: Coronary artery disease.

**A**

**
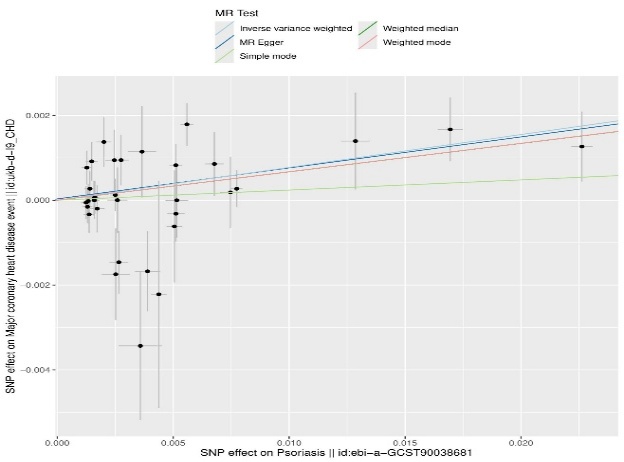
**

**B**

**
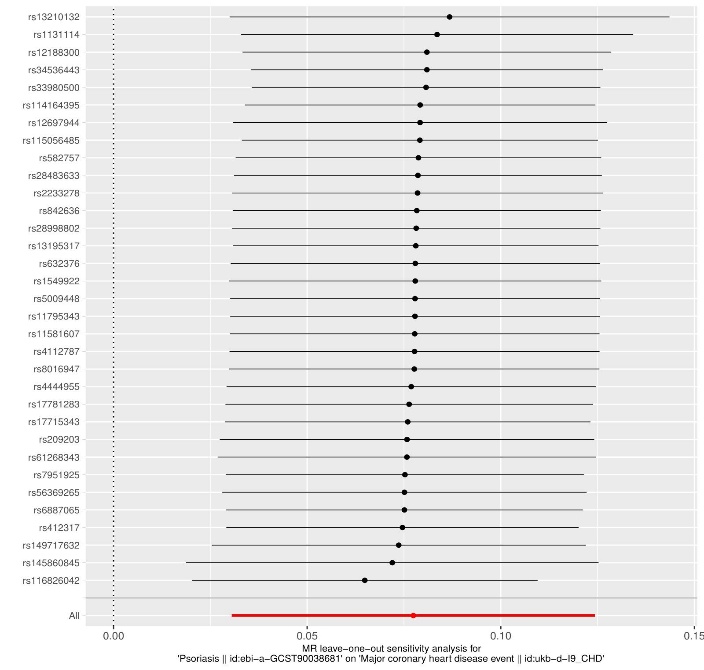
**

**C**

**
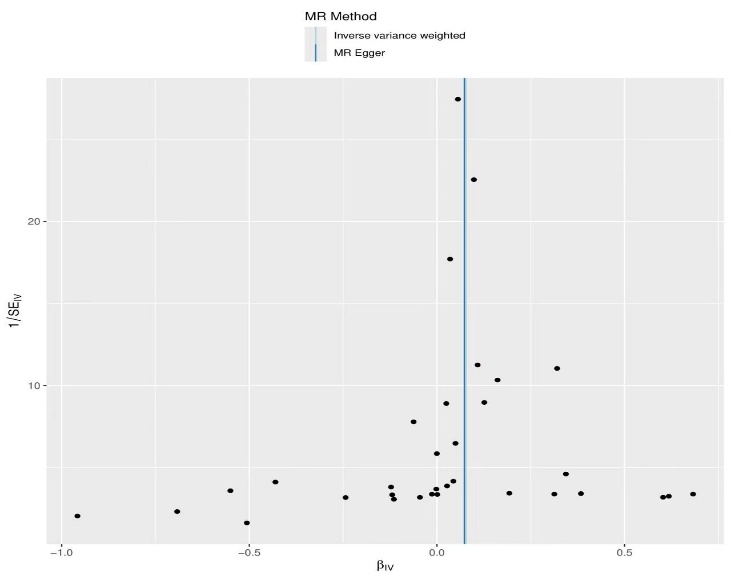
**

**Figure S2.** Scatter plots (A), Leave-one-out analysis (B) and Funnel plots (C) of psoriasis on CHD.

MR: Mendelian randomization; SNP: Single Nucleotide Polymorphism; CHD: Coronary heart disease.

**A**

**
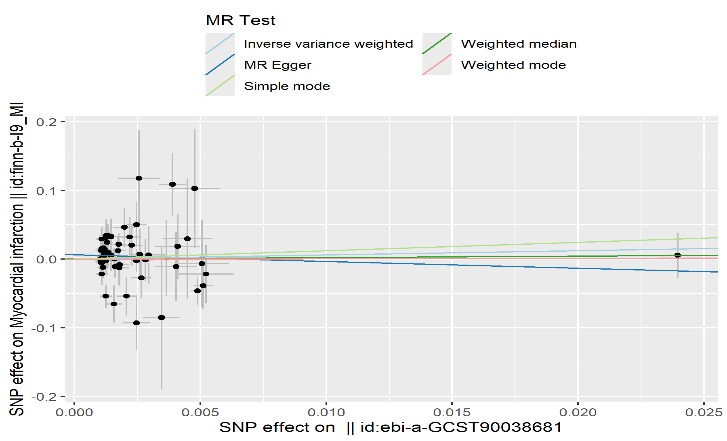
**

**B**

**
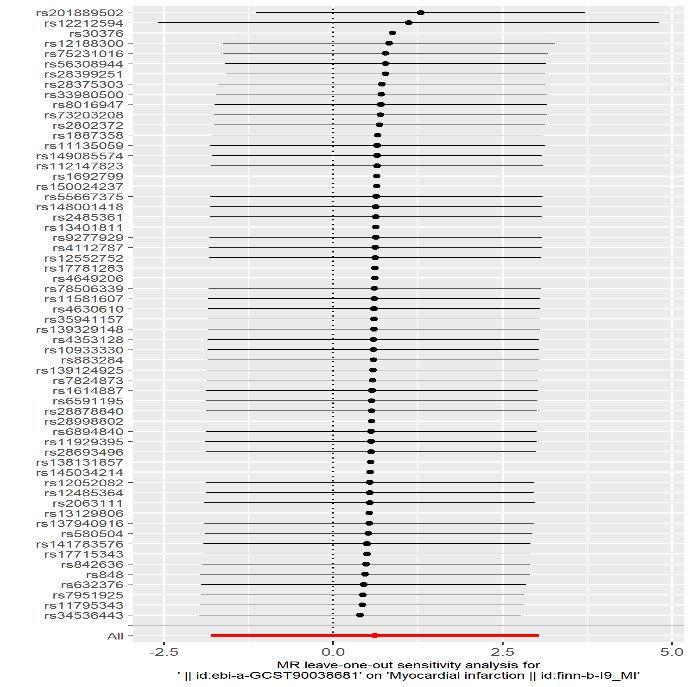
**

**C**

**
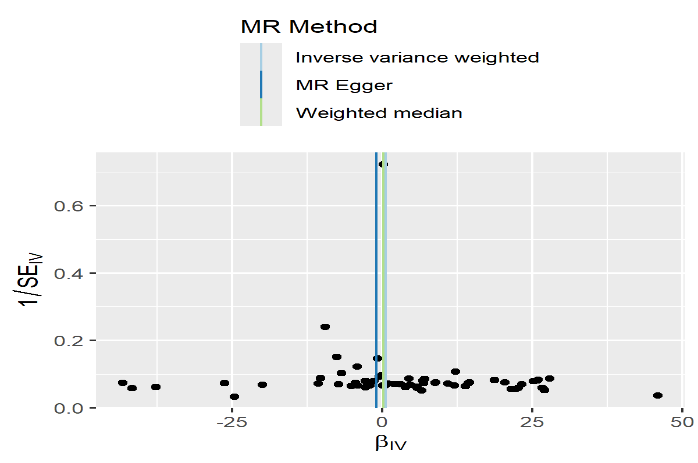
**

**Figure S3.** Scatter plots (A), Leave-one-out analysis (B) and Funnel plots (C) of psoriasis on MI.

MR: Mendelian randomization; SNP: Single Nucleotide Polymorphism; MI: myocardial infarction.

A


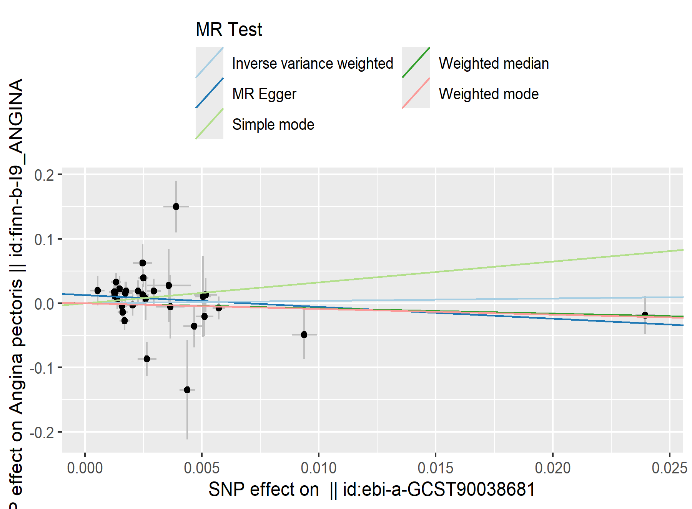


B


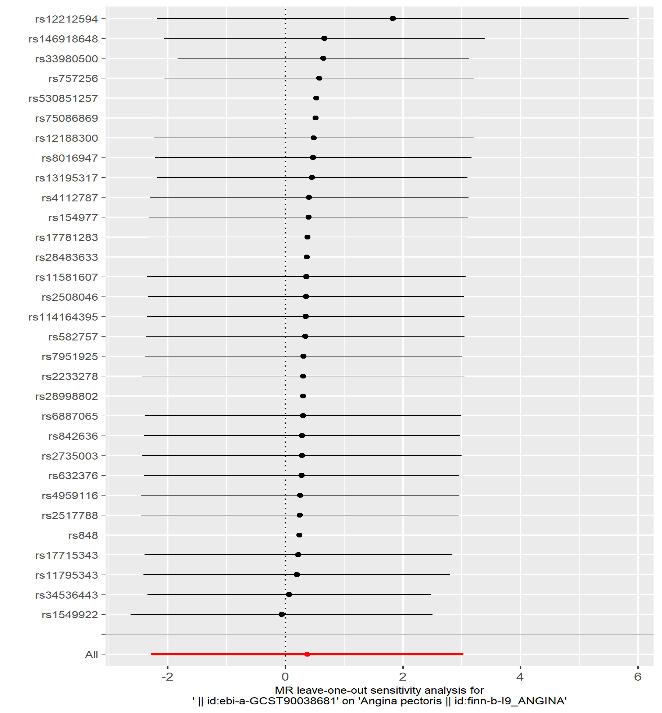


C


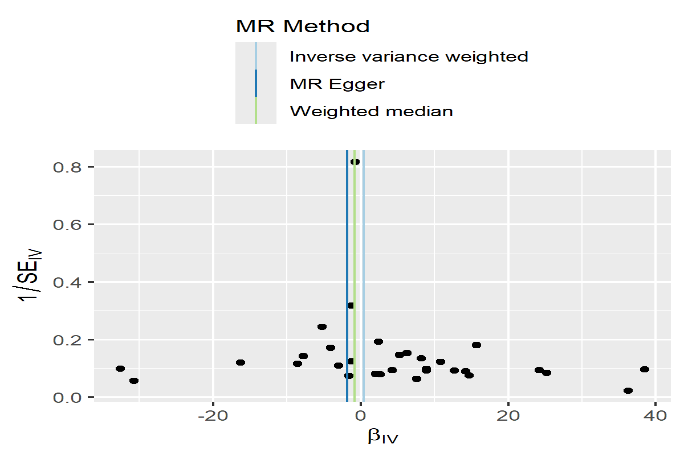


**Figure S4.** Scatter plots (A), Leave-one-out analysis (B) and Funnel plots (C) of psoriasis on angina pectoris. MR: Mendelian randomization; SNP: Single Nucleotide Polymorphism.

A


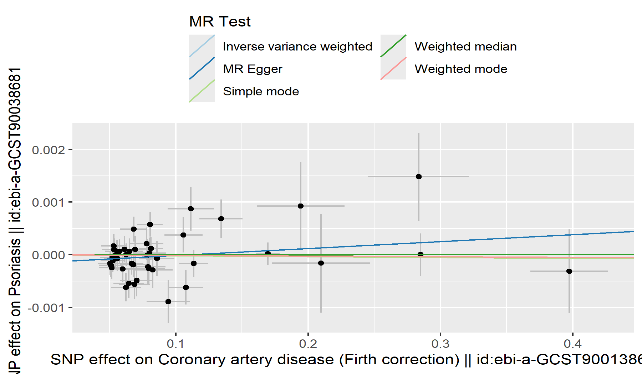


B


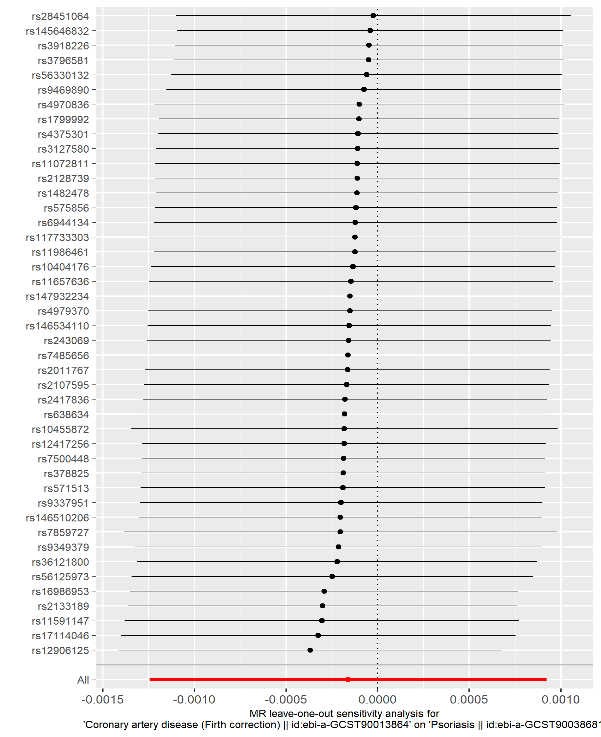


C


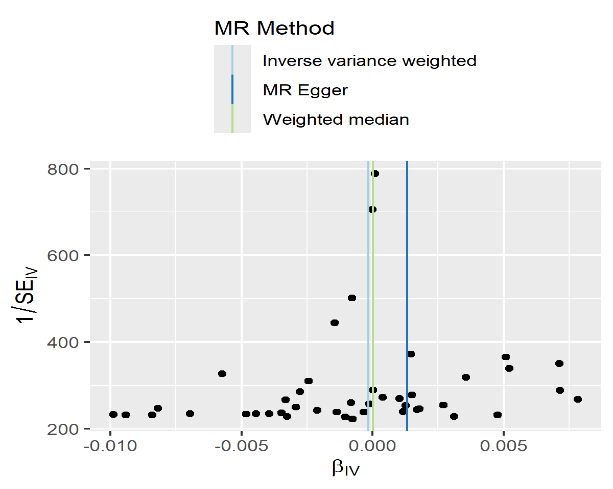


**Figure S5.** Scatter plots (A), Leave-one-out analysis (B) and Funnel plots (C) of CAD on psoriasis.

MR: Mendelian randomization; SNP: Single Nucleotide Polymorphism; CAD: Coronary artery disease.

**A**


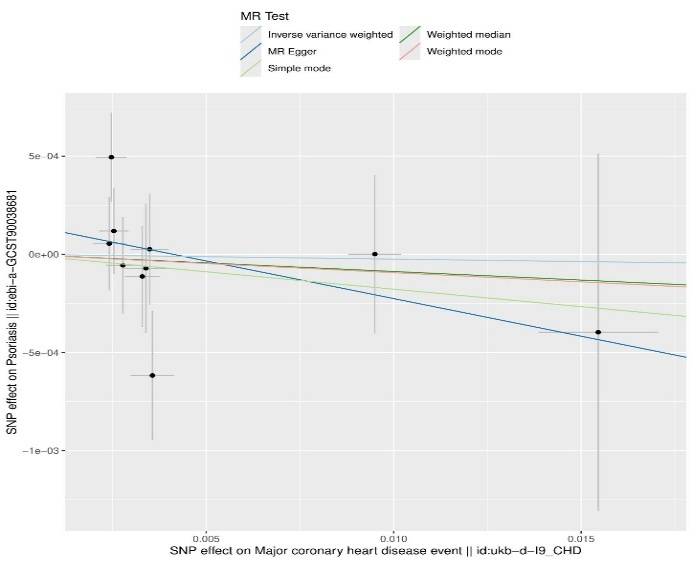


**B**

**
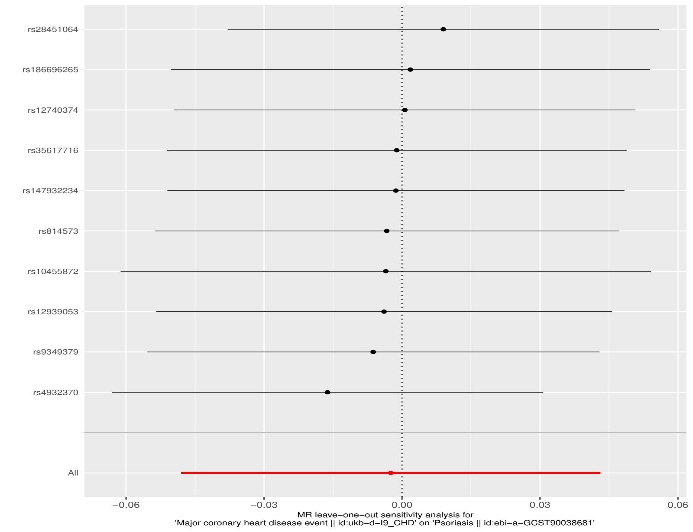
**

**C**

**
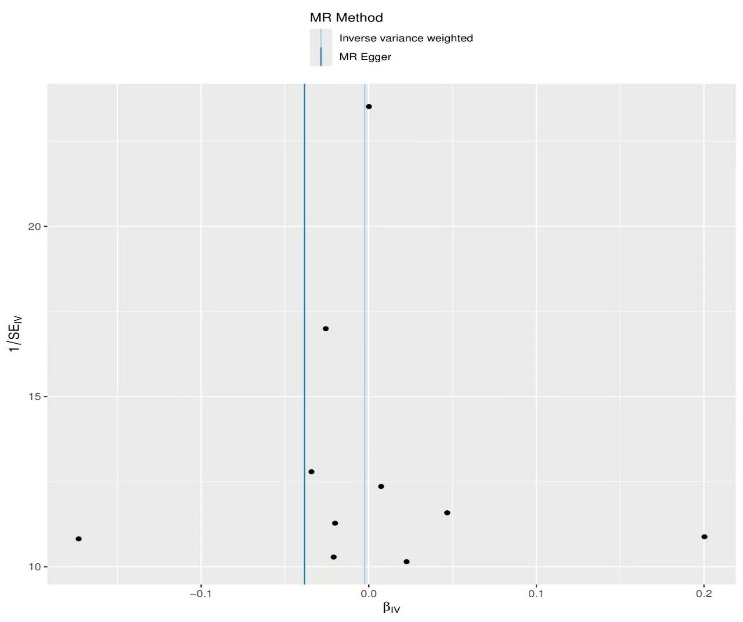
**

**Figure S6.** Scatter plots (A), Leave-one-out analysis (B) and Funnel plots (C) of CHD on psoriasis.

MR: Mendelian randomization; SNP: Single Nucleotide Polymorphism; CHD: Coronary heart disease.

A


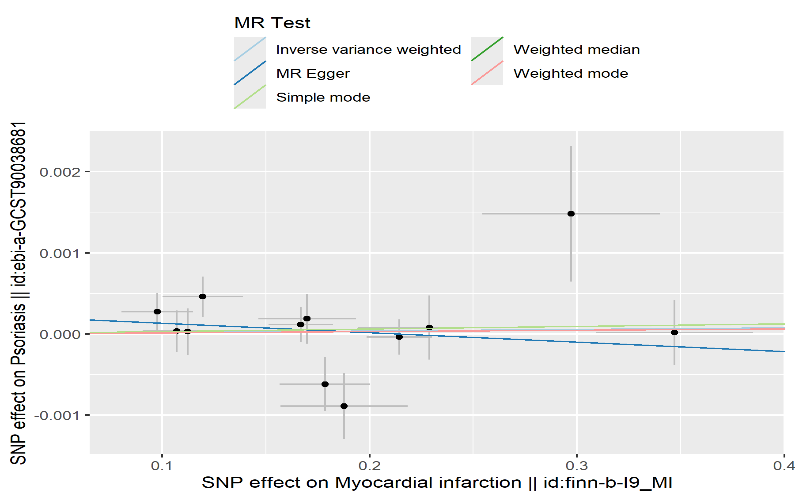


B


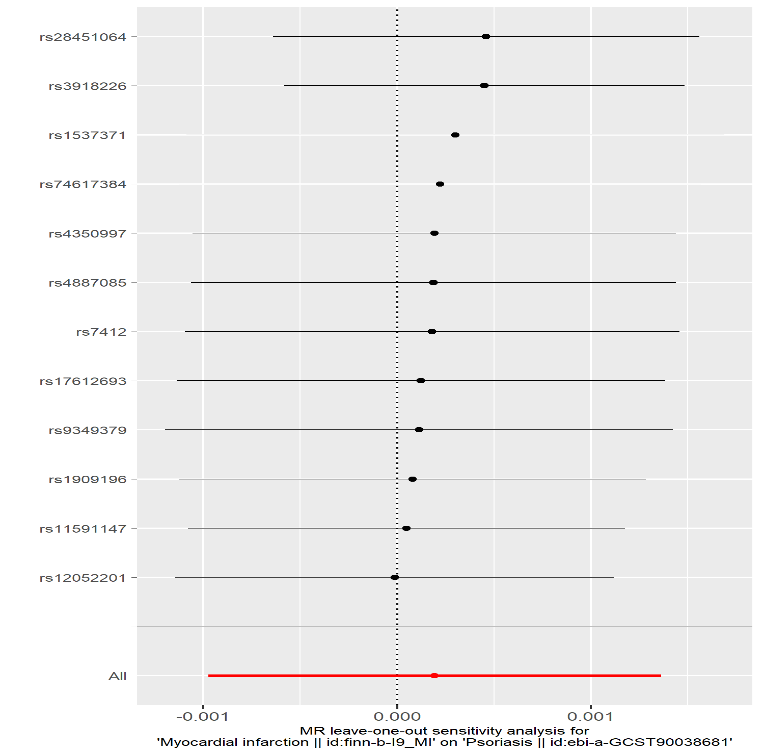


C


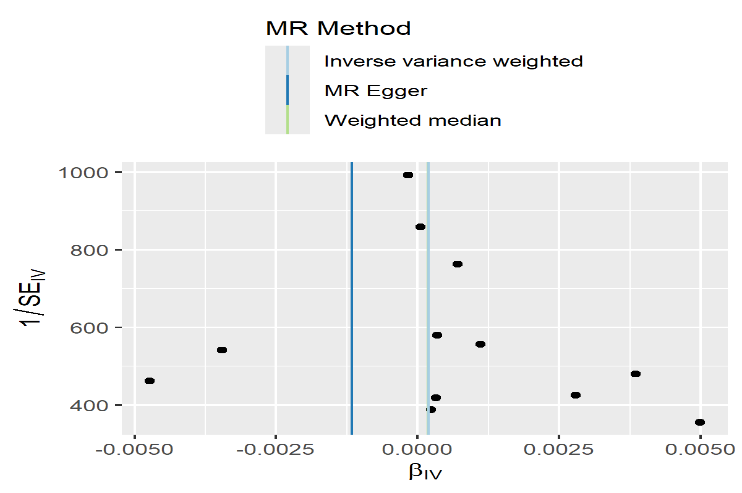


**Figure S7.** Scatter plots (A), Leave-one-out analysis (B) and Funnel plots (C) of MI on psoriasis.

MR: Mendelian randomization; SNP: Single Nucleotide Polymorphism; MI: myocardial infarction.

A


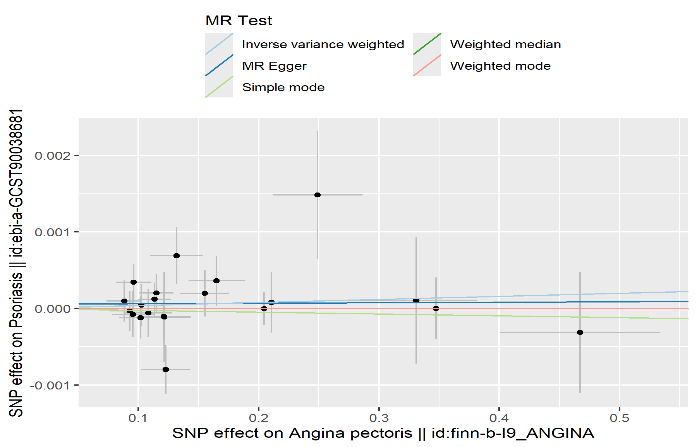


B


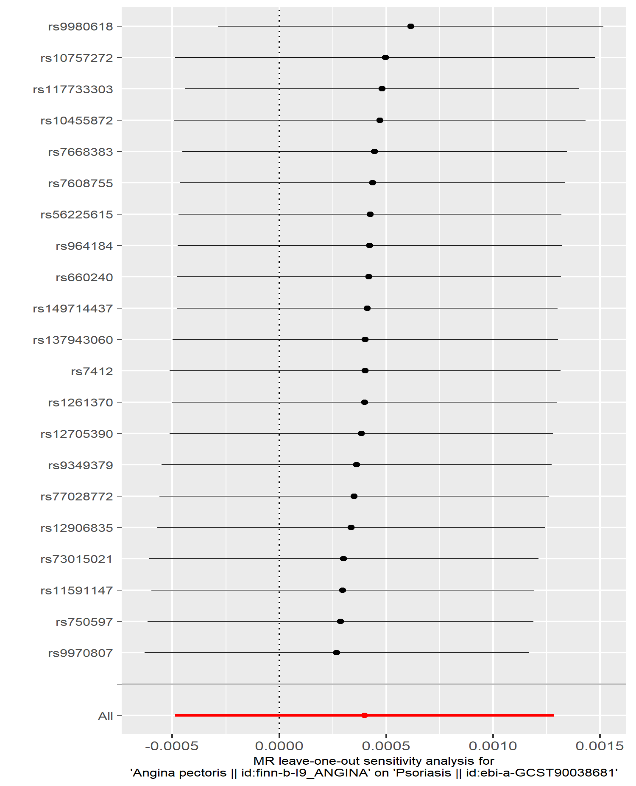


C

**
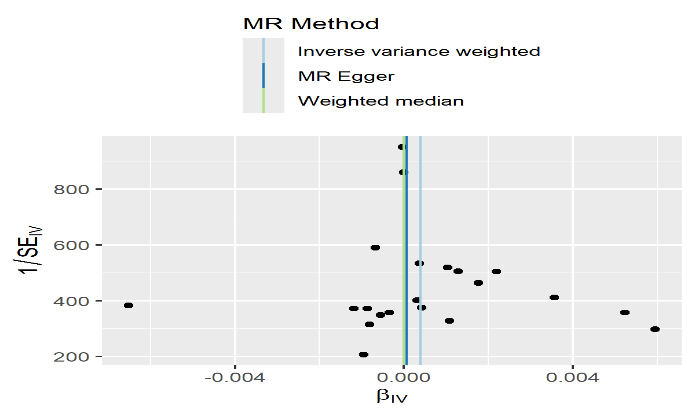
**

**Figure S8.** Scatter plots (A), Leave-one-out analysis (B) and Funnel plots (C) of angina pectoris on psoriasis. MR: Mendelian randomization; SNP: Single Nucleotide Polymorphism.
